# DNA Methylation Profiles of Ovarian Epithelial Carcinoma Tumors and Cell Lines

**DOI:** 10.1371/journal.pone.0009359

**Published:** 2010-02-22

**Authors:** Sahar Houshdaran, Sarah Hawley, Chana Palmer, Mihaela Campan, Mari N. Olsen, Aviva P. Ventura, Beatrice S. Knudsen, Charles W. Drescher, Nicole D. Urban, Patrick O. Brown, Peter W. Laird

**Affiliations:** 1 Department of Biochemistry and Molecular Biology, University of Southern California, Los Angeles, California, United States of America; 2 Canary Foundation, Palo Alto, California, United States of America; 3 Department of Surgery, University of Southern California, Los Angeles, California, United States of America; 4 Department of Biochemistry, Stanford University, Stanford, California, United States of America; 5 Fred Hutchinson Cancer Research Center, Seattle, Washington, United States of America; 6 University of Southern California Epigenome Center, University of Southern California, Los Angeles, California, United States of America; Deutsches Krebsforschungszentrum, Germany

## Abstract

**Background:**

Epithelial ovarian carcinoma is a significant cause of cancer mortality in women worldwide and in the United States. Epithelial ovarian cancer comprises several histological subtypes, each with distinct clinical and molecular characteristics. The natural history of this heterogeneous disease, including the cell types of origin, is poorly understood. This study applied recently developed methods for high-throughput DNA methylation profiling to characterize ovarian cancer cell lines and tumors, including representatives of three major histologies.

**Methodology/Principal Findings:**

We obtained DNA methylation profiles of 1,505 CpG sites (808 genes) in 27 primary epithelial ovarian tumors and 15 ovarian cancer cell lines. We found that the DNA methylation profiles of ovarian cancer cell lines were markedly different from those of primary ovarian tumors. Aggregate DNA methylation levels of the assayed CpG sites tended to be higher in ovarian cancer cell lines relative to ovarian tumors. Within the primary tumors, those of the same histological type were more alike in their methylation profiles than those of different subtypes. Supervised analyses identified 90 CpG sites (68 genes) that exhibited ‘subtype-specific’ DNA methylation patterns (FDR<1%) among the tumors. In ovarian cancer cell lines, we estimated that for at least 27% of analyzed autosomal CpG sites, increases in methylation were accompanied by decreases in transcription of the associated gene.

**Significance:**

The significant difference in DNA methylation profiles between ovarian cancer cell lines and tumors underscores the need to be cautious in using cell lines as tumor models for molecular studies of ovarian cancer and other cancers. Similarly, the distinct methylation profiles of the different histological types of ovarian tumors reinforces the need to treat the different histologies of ovarian cancer as different diseases, both clinically and in biomarker studies. These data provide a useful resource for future studies, including those of potential tumor progenitor cells, which may help illuminate the etiology and natural history of these cancers.

## Introduction

Ovarian cancer is the leading cause of death among all gynecological cancers in the United States [1], and is the sixth leading cause of all cancer deaths among women. There are four major histological types of ovarian cancer (serous, endometrioid, mucinous, and clear cell), each with distinct histopathological, clinical and molecular characteristics. The natural history of ovarian tumors, including their cell type of origin and steps of carcinogenesis, is poorly understood and has been the subject of much debate and discussion. Many studies of ovarian cancer are based on ovarian cancer derived cell lines, but it is unclear to what extent these cell lines accurately model the disease with respect to various molecular characteristics, including DNA methylation status.

DNA methylation is one of the epigenetic mechanisms that plays a role in many important biological processes including X-inactivation [2]–[4], silencing parasitic DNA elements [5], genomic imprinting [6], aging [7], male infertility [8], and cancer. Previous studies have shown CpG island DNA hypermethylation in various cancers, including ovarian tumors, as well as reduced levels of global DNA methylation associated with cancer [9]–[15]. Furthermore, the DNA methylation profile of a tumor cell is a reflection of its somatic lineage, environmental exposure, genetic predisposition, and cell-type specific chromatin structure. Recent technological advances have enabled quantitative assessment of the DNA methylation status of thousands of loci at once, providing an unprecedented opportunity to investigate the epigenetic signature of a cell. This rich source of information could be utilized to better understand the cell of origin of the subtypes and the steps of carcinogenesis, to identify appropriate model systems for future studies, and to discover candidate biomarkers for disease detection, classification, and monitoring.

## Results

### DNA Methylation Profiles of Ovarian Surface Epithelial Primary Tumors and Cell Lines

We assessed the DNA methylation profile of 1,505 CpG sites (associated with 808 genes) in 15 ovarian cell lines and 27 primary tumors (15 serous, 9 endometrioid, and 3 clear cell), using the Illumina GoldenGate Cancer Panel I [16] (see Supplemental File S1 for methylation array details). This panel has been used in several previous studies by others and us [8], [16]–[22]. Several investigators have validated the DNA methylation results of this panel by pyrosequencing [17], [19], Methylation Sensitive PCR (MSP) [16], [21], and bisulfite genomic sequencing [16]. The reproducibility of this assay has also been reported previously [16], [19]. The clinical and histopathological characteristics of the tumors and cell lines are detailed in Tables 1 and 2 respectively.

**Table 1:** Histology and clinical characteristics of primary ovarian tumors.

Tumor No.	Histology	Stage	PatientAge	CA125 (U/mL)	Menopausal Status	Elevated Risk
CC-T1	Clear Cell	IA	51	25	Peri	N
CC-T2	Clear Cell	IC	58	24	Post	N
CC-T3	Clear Cell	IC	45	705	Peri	N
E-T1	Endometrioid	IIIC	75	112	Post	N
E-T2	Endometrioid	IIB	43	207	Pre	N
E-T3	Endometrioid	IIIC	58	2662	Post	Y
E-T4	Endometrioid	IIA	58	75	Post	Y
E-T5	Endometrioid	IA	55	372	Post	N
E-T6	Endometrioid	IB	46	3897	Peri	N
E-T7	Endometrioid	IB	47	5201	Post	N
E-T8	Endometrioid	IC	38	1720	Post	Y
E-T9	Endometrioid	IIIB	51	2301	Post	N
S-T1	Serous	IIIC	82	134	Post	N
S-T2	Serous	IVA	58	6706	Post	N
S-T3	Serous	IIIC	77	1828	Post	N
S-T4	Serous	IIIC	74	994	Post	N
S-T5	Serous	IIIC	43	480	Pre	N
S-T6	Serous	IIIC	70	1504	Post	N
S-T7	Serous	IIIC	74	1023	Post	Y
S-T8	Serous	IIIC	56	396	Post	N
S-T9	Serous	IIIC	69	835	Post	N
S-T10	Serous	IIIC	59	4626	Post	N
S-T11	Serous	IIIC	48	12	Post	Y
S-T12	Serous	IIIC	68	49	Post	N
S-T13	Serous	IIIC	49	195	Peri	N
S-T14	Serous	IIIC	42	1075	Pre	N
S-T15	Serous	IIIC	49	8000	Post	N

Menopausal Status was recorded as follows: Post  =  age> =  55 or self-reported menopause, hormone replacement therapy, hysterectomy or oophorectomy; Peri  =  age<55 and inconsistent menstruation; Pre  =  age<55 and menstruating (See Appendix S1). Elevated Risk was recorded as follows: Y if one or more of the following conditions were met: a) family history of ovarian or breast cancer; b) Ashkenazi Jewish ethnicity and family history of breast cancer; c) BRCA1 or 2 mutation; d) mutation status unknown and family history of BRCA1 or 2 mutation [64].

**Table 2:** Characteristics of ovarian cancer cell lines.

Cell Line	Histology	Derivation Notes	Ref.	Source
2008	Serous	unknown - serous - stage IV	[65]	Dr. George Coukos (Abramson Family Cancer Research Institute at University of Pennsylvania)
A1847	Adenocarcinoma	unknown	[66]	Dr. Ingegerd Hellström (University of Washington, Seattle)
A2780	Adenocarcinoma	tumor	[67]	Dr. Tom Hamilton (Fox Chase Cancer Research Center, Philadelphia)
CAOV3	Adenocarcinoma	tumor	ATCC (J. Fogh)	American Type Culture Collection (HTB-75)
ES2	Clear Cell	tumor - clear cell	[68]	American Type Culture Collection (CRL-1978)
IGROV1	Endometrioid	tumor - endometrioid - stage III	[69]	Pacific Ovarian Cancer Research Consortium
HEY	Serous	tumor - serous	[70]	Dr. Naoto Ueno (M.D. Anderson Cancer Center)
OVCAR3	Serous	ascites - adenocarcinoma	[71]	Pacific Ovarian Cancer Research Consortium
OVCAR5	Adenocarcinoma	tumor	[72]	Dr. Ingegerd Hellström (University of Washington, Seattle)
OVCAR10	Adenocarcinoma	tumor	[72]	Dr. Ingegerd Hellström (University of Washington, Seattle)
OV90	Serous	ascites - serous - stage IIIC - grade 3	[73]	American Type Culture Collection (CRL-11732)
PEO1	Adenocarcinoma	ascites - stage III	[74]	Dr. Ingegerd Hellström (University of Washington, Seattle)
SKOV3	Adenocarcinoma	ascites	[75]	American Type Culture Collection (HTB-77)
TOV112D	Endometrioid	tumor - endometrioid - stage IIIC- grade 3	[73]	American Type Culture Collection (CRL-11731)
TOV21G	Clear Cell	tumor - clear cell - stage III - grade 3	[73]	American Type Culture Collection (CRL-11730)

**Figure 1:**
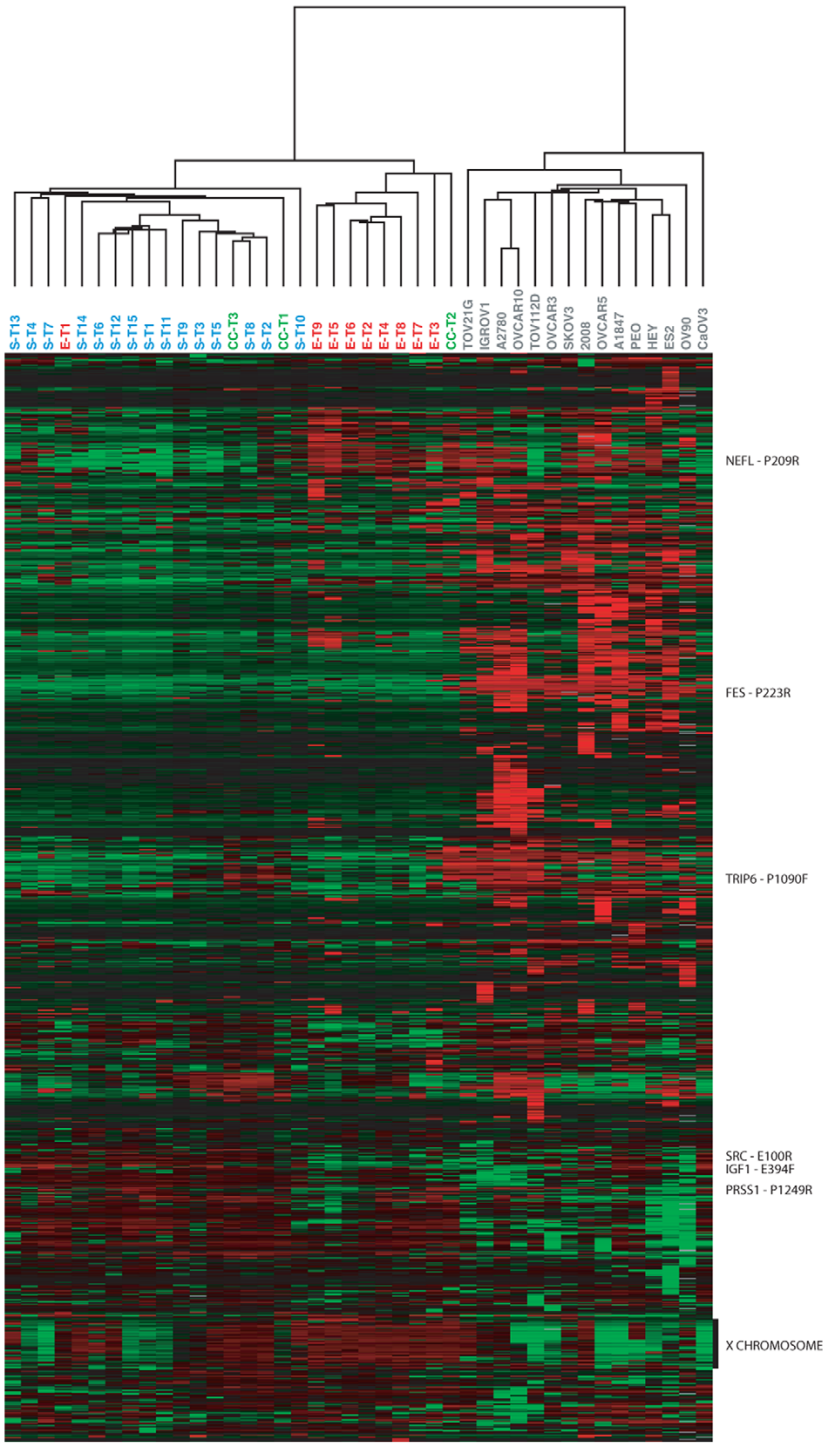
DNA methylation profiles of ovarian cancer cell lines and tumors. DNA methylation profiles for 27 ovarian primary tumors and 15 ovarian cell lines are shown. Only the 1,184 CpG sites (686 genes) with variable DNA methylation levels across these specimens are shown (see Methods). Methylation values (β-values) were mean centered for each gene (across all specimens) then CpG sites and specimens were hierarchically clustered. Red indicates high methylation relative to the site-specific mean, green indicates low methylation relative to the mean. Rows of data corresponding to selected CpG sites (those with the greatest magnitude of difference between tumors and cell lines, or between pairs of tumor histologies) are indicated to the right of the panel. **CL** = Cell line, **S** = Serous tumor, **E** = Endometrioid tumor, **CC** = Clear cell tumor.

Of the 1,505 CpG sites (808 genes) surveyed, 1,184 sites (686 genes) exhibited sufficient variation across the 42 specimens for further analysis (see Methods). Unsupervised hierarchical clustering of both specimens and CpG sites using these 1,184 probes is shown in Figure 1. The primary division in the clustering of the specimens was between cell lines and tumors, each of which formed a distinct cluster. Among the tumors, the primary serous and endometrioid tumors each formed a distinct cluster (with one exception), whereas the three clear cell tumors were distributed between the two main clusters. There was no clear pattern of sub-clustering among the cell lines, despite the diversity of histological types nominally represented. The most notable cluster of CpG sites was a large group of CpG sites on the X chromosome with highly correlated methylation levels across all specimens. Because of the special role of DNA methylation on the X chromosome in dosage compensation, we excluded X-linked genes from further analyses. DNA methylation measurements from multiple probes representing different CpG sites associated with the same gene tended to be highly correlated (mean r value: 0.64 for related pairs of probes and 0.04 for unrelated pairs; see Figures S1 and S2).

**Figure 2:**
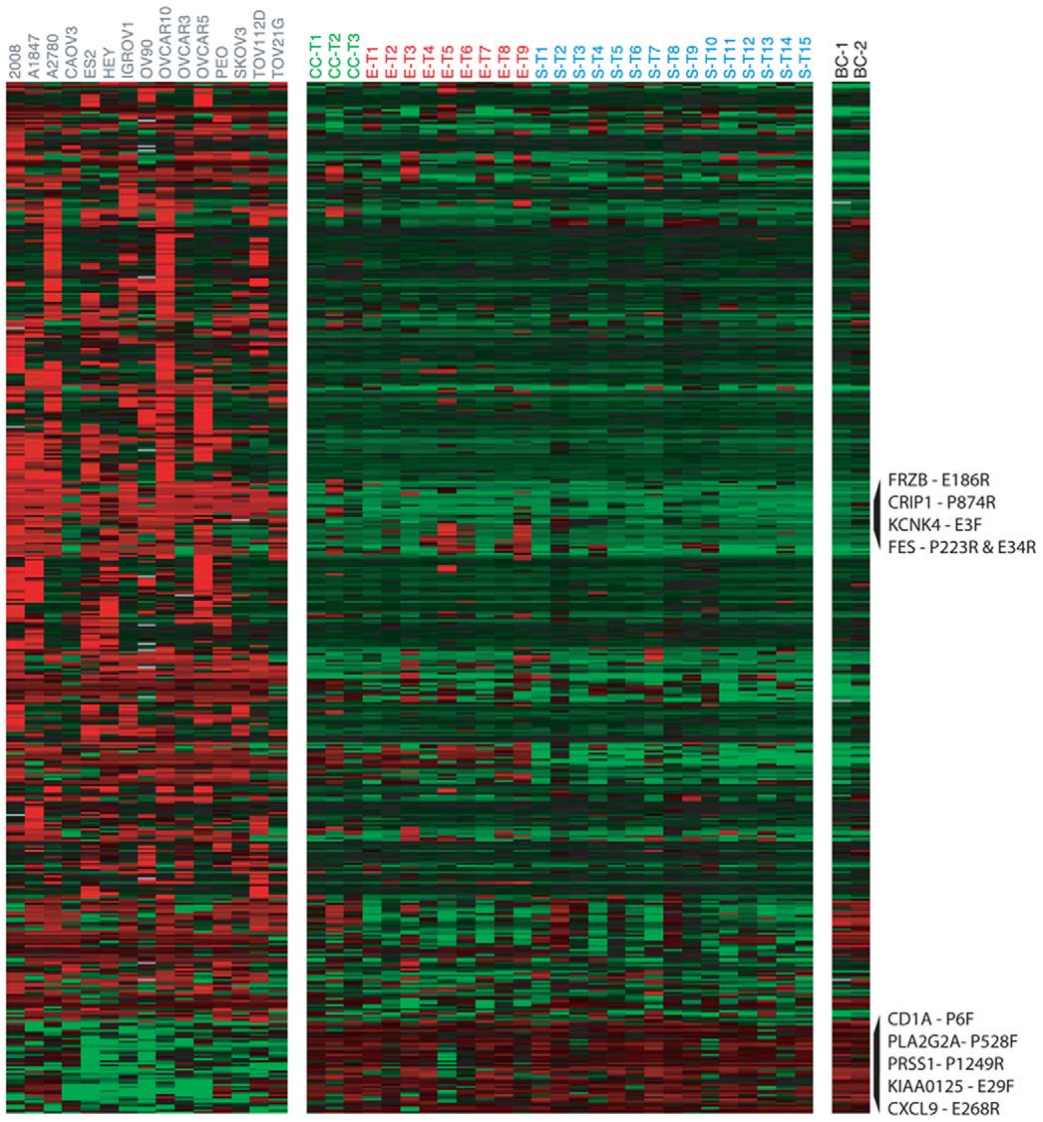
Differential DNA methylation between ovarian cell lines and tumors. DNA methylation levels in tumors, cell lines and buffy coat (leukocytes) are shown for the 489 autosomal CpG sites (337 genes) with significant differences in DNA methylation between cell lines and tumors. Methylation values (β-values) were mean centered for each gene (across tumor and cell line specimens). Data were hierarchically clustered only for the CpG sites. The displayed CpG sites were selected based on the adjusted p-values associated with differential expression between tumors and cell lines. Red indicates high methylation relative to the site-specific mean, green indicates low methylation relative to the mean. Rows of data corresponding to sites with the greatest magnitude of difference in methylation levels (β-values) between tumors and cell lines are labeled (top 5 sites in each direction). **CL** =  Cell line, **S** = Serous tumor, **E** = Endometrioid tumor, **CC** = Clear cell tumor, **BC** = Buffy coat.

We further explored differences in DNA methylation between cell lines and tumors, using a two-sample t-test to compare DNA methylation levels of selected CpG sites. Only the 1,110 CpG sites that met a minimal level of variability in methylation levels across these specimens (see Methods) were included in this analysis. We thus identified 489 CpG sites (associated with 337 genes) that varied significantly in DNA methylation level between the ovarian cancer derived cell lines and the primary ovarian cancer tumors at a false discovery rate (FDR) less than 1% [23] (see Figure 2 and Supplemental File S2). Among CpG sites whose mean methylation levels differed between cell lines and tumors, the vast majority were more highly methylated in the cell lines (445 of 489 CpG sites, 299 of 337 genes). The proposed cell of origin of ovarian carcinoma (Ovarian Surface Epithelium) is currently under debate [24]. Whole ovary, which is comprised of various cell types, including granulosa and follicular cells, is not an appropriate control. On the other hand, the normal cells that contaminate tumor samples include blood cells. Therefore, we compared DNA methylation levels in leukocytes (obtained from buffy coat) from two healthy females (age>60). The inclusion of leukocytes could also serve as a first step to assess the feasibility of blood-based biomarker development. For the majority of probes, DNA methylation levels in the leukocytes were similar to those observed in the tumor samples (see Figure 2). The elevated levels of DNA methylation in the leukocytes among the CpG sites that had elevated methylation in tumors relative to cell lines was particularly striking. The 39 CpG sites (associated with 35 genes) whose methylation levels differed most significantly between cell lines from tumors (FDR<0.1%) and had the largest differences in mean methylation levels between cell lines and tumors are shown in Table 3.

**Table 3:** Loci with differential methylation between ovarian tumors and cell lines.

Gene Symbol	Probe ID	Cell Line-Tumor (mean β)	Cell Lines (mean β)	Tumors (mean β)
**CL > TUMORS**				
FES	FES_P223_R	0.79	0.87	0.08
FRZB	FRZB_E186_R	0.77	0.95	0.19
FES	FES_E34_R	0.76	0.84	0.08
KCNK4	KCNK4_E3_F	0.75	0.89	0.14
CRIP1	CRIP1_P874_R	0.75	0.90	0.15
POMC	POMC_P400_R	0.75	0.87	0.12
TBX1	TBX1_P885_R	0.72	0.91	0.19
COL1A2	COL1A2_P48_R	0.68	0.88	0.20
FRZB	FRZB_P406_F	0.67	0.74	0.06
PYCARD	PYCARD_E87_F	0.66	0.75	0.09
PROK2	PROK2_P390_F	0.65	0.66	0.01
ESR2	ESR2_E66_F	0.65	0.66	0.01
DES	DES_E228_R	0.64	0.77	0.13
PODXL	PODXL_P1341_R	0.63	0.71	0.08
THBS2	THBS2_P605_R	0.62	0.83	0.21
TNFRSF10C	TNFRSF10C_E109_F	0.62	0.83	0.21
COL1A2	COL1A2_E299_F	0.61	0.82	0.21
FGF8	FGF8_P473_F	0.61	0.79	0.17
KIT	KIT_P367_R	0.60	0.67	0.06
COL1A1	COL1A1_P5_F	0.60	0.74	0.14
**TUMORS > CL**				
PRSS1	PRSS1_P1249_R	−0.58	0.23	0.81
PLA2G2A	PLA2G2A_P528_F	−0.56	0.23	0.79
KIAA0125	KIAA0125_E29_F	−0.54	0.19	0.73
CD1A	CD1A_P6_F	−0.51	0.29	0.80
CXCL9	CXCL9_E268_R	−0.45	0.28	0.73
GABRG3	GABRG3_P75_F	−0.44	0.38	0.83
PLA2G2A	PLA2G2A_E268_F	−0.43	0.46	0.89
USP29	USP29_E274_F	−0.42	0.44	0.85
CTLA4	CTLA4_P1128_F	−0.41	0.41	0.82
GABRG3	GABRG3_E123_R	−0.40	0.51	0.92
GML	GML_P281_R	−0.39	0.45	0.84
PTHR1	PTHR1_P258_F	−0.38	0.30	0.68
GNAS	GNAS_E58_F	−0.34	0.53	0.86
CHI3L2	CHI3L2_P226_F	−0.30	0.57	0.86
DSG1	DSG1_P159_R	−0.30	0.63	0.92
IL4	IL4_P262_R	−0.29	0.61	0.90
BMPR1A	BMPR1A_E88_F	−0.27	0.57	0.83
LMTK2	LMTK2_P1034_F	−0.26	0.51	0.77

The 39 CpG sites (35 genes) with the greatest difference in DNA methylation levels between cell lines and tumors are shown. Probes were ranked first by significance (FDR<0.1%) then by magnitude of mean difference between these two groups.

**Figure 3:**
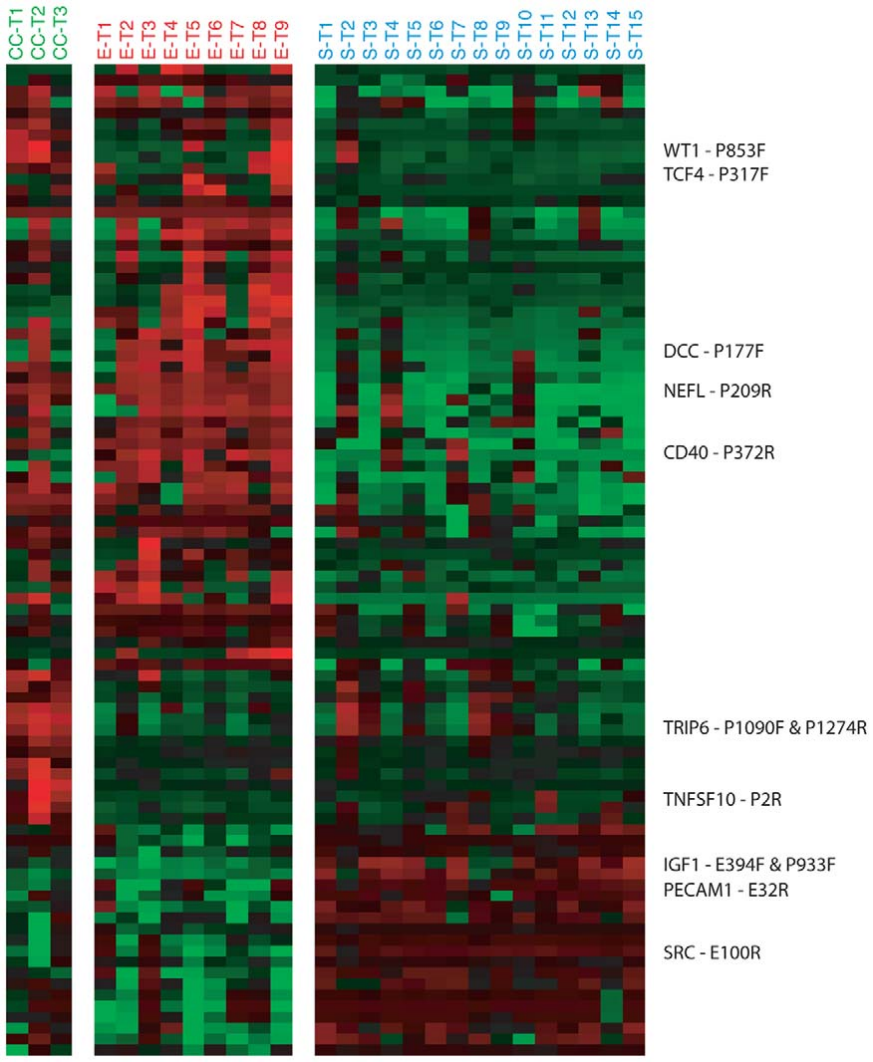
Differential DNA methylation among ovarian tumor histologies. DNA methylation levels are shown for the 90 autosomal CpG sites (68 genes) with a significant difference in methylation between one or more pairs of tumor histologies. Methylation values (β-values) were mean centered for each gene (across tumors). Data were hierarchically clustered only for the CpG sites. The displayed CpG sites were selected based on the adjusted p-values associated with differential expression between tumors and cell lines. Red indicates high methylation relative to the site-specific mean, green indicates low methylation relative to the mean. Rows of data corresponding to sites with the greatest magnitude of difference in methylation levels (β-values) between pairs of tumor histologies are labeled (up to 3 sites in each direction for each pairwise comparison). **S** = Serous tumor, **E** = Endometrioid tumor, **CC** = Clear cell tumor.

To explore differences in DNA methylation profiles between tumor histologies, we compared the DNA methylation levels of 818 autosomal CpG sites selected for variability in DNA methylation levels across the tumors (see Methods). We applied a two-sample t-test to compare pairs of tumor histologies, and adjusted for multiple comparisons by controlling the false discovery rate [23] (see Methods for details). In this way, we identified 90 CpG sites (68 genes) that showed significant differences in methylation levels between at least one pair of tumor histologies at an FDR<1% (see Figure 3, Supplemental File S2). Note that a small fraction of the CpG sites with ‘subtype-specific’ DNA methylation were identified in more than one pairwise comparison (11 of 90 CpG sites; 12 of 68 genes). Serous and endometrioid tumors were distinguished by the largest number of CpG sites (70 CpG sites, 52 genes), of which 49 CpG sites (36 genes) were more highly methylated on average in endometrioid tumors. Nineteen CpG sites (18 genes) and 12 CpG sites (10 genes) differed significantly in mean methylation between clear cell and serous tumors, or clear cell and endometrioid tumors, respectively. Nearly all of the 26 CpG sites (23 genes) with differential methylation between clear cell tumors and serous or endometrioid tumors (or both), were more highly methylated on average in clear cell tumors (24 of 26 CpG sites, 21 of 23 genes). For each pairwise comparison, the CpG sites with the most significant differences in methylation level between the two tumor histologies are shown in Tables 4, 5 and 6 (see Supplemental Figure S3 for extent of overlap between CpG sites and genes across lists, and Supplemental File S3, for CpG island information and distance to transcription start site for the probes shown in Tables 4, 5 and 6).

**Table 4:** Loci with differential methylation between endometrioid versus serous tumors (FDR<0.1%).

Gene Symbol	Probe ID	Endometrioid- Serous (mean β)	Endometrioid (mean β)	Serous (mean β)	Clear Cell (mean β)
**ENDO > SEROUS**					
NEFL	NEFL_P209_R	0.69	0.90	0.21	0.88
CD40	CD40_P372_R	0.62	0.77	0.15	0.22
DCC	DCC_P177_F	0.59	0.68	0.08	0.35
NEFL	NEFL_E23_R	0.57	0.80	0.23	0.68
PENK	PENK_P447_R	0.56	0.82	0.26	0.78
ADCYAP1	ADCYAP1_P398_F	0.55	0.69	0.14	0.28
LMO2	LMO2_P794_R	0.54	0.98	0.44	0.97
JAK3	JAK3_P156_R	0.53	0.91	0.37	0.77
HTR1B	HTR1B_P222_F	0.51	0.74	0.23	0.51
TMEFF2	TPEF_seq_44_S88_R	0.50	0.66	0.16	0.58
DCC	DCC_P471_R	0.50	0.64	0.15	0.29
ISL1	ISL1_P379_F	0.49	0.58	0.09	0.06
HPN	HPN_P374_R	0.48	0.69	0.21	0.77
ISL1	ISL1_P554_F	0.48	0.54	0.06	0.29
EYA4	EYA4_P794_F	0.48	0.84	0.36	0.69
FGF2	FGF2_P229_F	0.46	0.71	0.25	0.40
TERT	TERT_P360_R	0.46	0.72	0.27	0.55
ISL1	ISL1_E87_R	0.45	0.50	0.05	0.07
DLK1	DLK1_E227_R	0.45	0.91	0.46	0.75
THY1	THY1_P149_R	0.45	0.81	0.37	0.67
JAK3	JAK3_E64_F	0.44	0.52	0.08	0.58
ADCYAP1	ADCYAP1_P455_R	0.42	0.53	0.10	0.50
HPN	HPN_P823_F	0.40	0.82	0.42	0.98
ZNF264	ZNF264_P397_F	0.37	0.94	0.57	0.63
PRKCDBP	PRKCDBP_P352_R	0.37	0.52	0.16	0.44
GUCY2D	GUCY2D_E419_R	0.36	0.41	0.04	0.05
FABP3	FABP3_E113_F	0.36	0.41	0.04	0.15
MLF1	MLF1_E243_F	0.29	0.43	0.14	0.17
ADCYAP1	ADCYAP1_E163_R	0.27	0.30	0.02	0.13
PYCARD	PYCARD_P150_F	0.22	0.86	0.64	0.73
SPARC	SPARC_E50_R	0.21	0.34	0.14	0.33
COMT	COMT_E401_F	0.17	0.30	0.13	0.19
THY1	THY1_P20_R	0.16	0.47	0.31	0.50
**SEROUS > ENDO**					
IGF1	IGF1_E394_F	−0.49	0.20	0.69	0.41
IGF1	IGF1_P933_F	−0.47	0.08	0.55	0.13
SRC	SRC_E100_R	−0.39	0.54	0.93	0.48
PI3	PI3_P1394_R	−0.39	0.39	0.78	0.54
ASB4	ASB4_P52_R	−0.38	0.58	0.96	0.88
BLK	BLK_P14_F	−0.38	0.42	0.80	0.55
JAK3	JAK3_P1075_R	−0.38	0.35	0.72	0.75
MOS	MOS_P746_F	−0.37	0.52	0.89	0.65
FGF7	FGF7_P44_F	−0.34	0.56	0.90	0.60
PECAM1	PECAM1_E32_R	−0.33	0.58	0.91	0.58
NOTCH4	NOTCH4_P938_F	−0.31	0.53	0.84	0.79
P2RX7	P2RX7_P597_F	−0.28	0.68	0.97	0.99
SERPINA5	SERPINA5_E69_F	−0.27	0.58	0.85	0.68
DNAJC15	DNAJC15_P65_F	−0.26	0.71	0.98	0.63
ASB4	ASB4_E89_F	−0.25	0.70	0.96	0.89
RARA	RARA_P1076_R	−0.19	0.72	0.91	0.85
AGXT	AGXT_P180_F	−0.17	0.81	0.97	0.72
SRC	SRC_P164_F	−0.14	0.81	0.96	0.75
ABCC2	ABCC2_P88_F	−0.14	0.85	0.98	0.74

CpG sites with the most significant differences in DNA methylation levels are shown for the pair-wise comparisons between endometrioid and serous tumors. Probes were first ranked by significance then by magnitude of mean difference between groups.

**Table 5:** Loci with differential methylation between clear cell versus serous tumors (FDR<1%).

Gene Symbol	Probe ID	Clear Cell – Serous (mean β)	Clear Cell (mean β)	Serous (mean β)	Endometrioid (mean β)
**CC > SEROUS**					
NEFL	NEFL_P209_R	0.66	0.88	0.21	0.90
TCF4	TCF4_P317_F	0.60	0.70	0.09	0.25
WT1	WT1_P853_F	0.58	0.64	0.07	0.21
HPN	HPN_P374_R	0.57	0.77	0.21	0.69
PYCARD	PYCARD_E87_F	0.57	0.59	0.03	0.04
FGFR3	FGFR3_P1152_R	0.55	0.74	0.19	0.26
P2RX7	P2RX7_P119_R	0.54	0.68	0.14	0.15
TNFSF10	TNFSF10_P2_R	0.51	0.64	0.12	0.03
CCND2	CCND2_P887_F	0.45	0.50	0.05	0.15
CEBPA	CEBPA_P1163_R	0.43	0.49	0.05	0.05
CCND2	CCND2_P898_R	0.40	0.45	0.05	0.28
GSTM2	GSTM2_E153_F	0.39	0.45	0.06	0.34
SPDEF	SPDEF_E116_R	0.34	0.46	0.12	0.08
NTSR1	NTSR1_P318_F	0.32	0.40	0.08	0.44
MCC	MCC_P196_R	0.27	0.27	0.01	0.28
HLA-DRA	HLA-DRA_P77_R	0.24	0.30	0.07	0.19
GP1BB	GP1BB_E23_F	0.21	0.30	0.10	0.09
**SEROUS > CC**					
SRC	SRC_E100_R	−0.45	0.48	0.93	0.54
PECAM1	PECAM1_E32_R	−0.33	0.58	0.91	0.58

CpG sites with the most significant differences in DNA methylation levels are shown for pair-wise comparisons between clear cell and serous tumors. Probes were first ranked by significance then by magnitude of mean difference between groups.

**Table 6:** Loci with differential methylation between clear cell versus endometrioid tumors (FDR<5%).

Gene Symbol	Probe ID	Clear Cell - Endometrioid (mean β)	Clear Cell (mean β)	Endometrioid (mean β)	Serous (mean β)
TRIP6	TRIP6_P1090_F	0.71	0.91	0.20	0.31
TRIP6	TRIP6_P1274_R	0.62	0.80	0.18	0.30
TNFSF10	TNFSF10_P2_R	0.60	0.64	0.03	0.12
IL1RN	IL1RN_P93_R	0.58	0.87	0.29	0.32
THBS2	THBS2_P605_R	0.54	0.62	0.08	0.21
P2RX7	P2RX7_P119_R	0.53	0.68	0.15	0.14
CEBPA	CEBPA_P1163_R	0.44	0.49	0.05	0.05
SPDEF	SPDEF_E116_R	0.38	0.46	0.08	0.12
HOXC6	HOXC6_P585_R	0.38	0.51	0.13	0.21
ESR1	ESR1_P151_R	0.36	0.43	0.07	0.15
SPDEF	SPDEF_P6_R	0.33	0.45	0.12	0.25
GP1BB	GP1BB_E23_F	0.21	0.30	0.09	0.10

CpG sites with the most significant differences in DNA methylation levels are shown for pair-wise comparisons between clear cell and endometrioid tumors. Probes were first ranked by significance then by magnitude of mean difference between groups.

Age-associated DNA hypermethylation is a well-established phenomenon [7]. Therefore, we examined age-associated methylation changes in the tumor specimens. After adjusting for histological type and controlling the false discovery rate [23], no CpG sites showed a significant association with age.

### Relationship between Gene Expression and DNA Methylation Status

**Figure 4:**
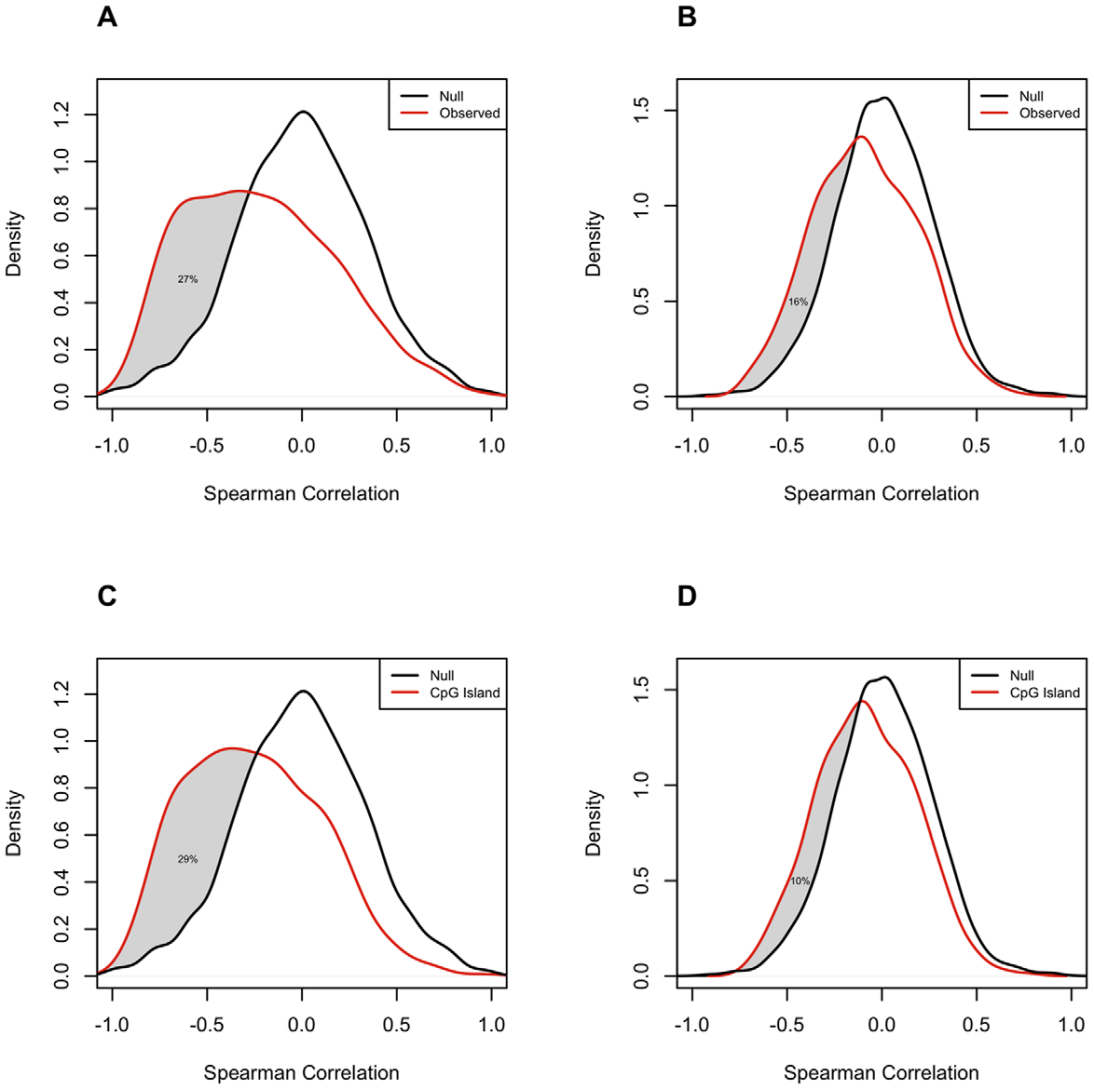
Correlations between DNA methylation and gene expression levels. The distributions of Spearman correlations between DNA methylation levels (β-values) and gene expression levels [log_2_(sample/reference)]. Cell lines and tumors were analyzed separately and was restricted to autosomal CpG sites with variable DNA methylation levels across the relevant specimen set and a minimum number of non-missing values (see Methods). A) Cell lines: Density plots of observed and simulated Spearman correlations for all filtered loci. N = 828 CpG sites (502 genes); B) Tumors: Density plots of observed and simulated Spearman correlations for all filtered loci. N = 758 CpG sites (482 genes); C) Cell Lines: Density plot of observed and simulated Spearman correlations for loci meeting CpG island criteria. N = 585; D) Tumors: Density plot of observed and simulated Spearman correlations for loci meeting CpG island criteria. N = 492.

**Figure 5:**
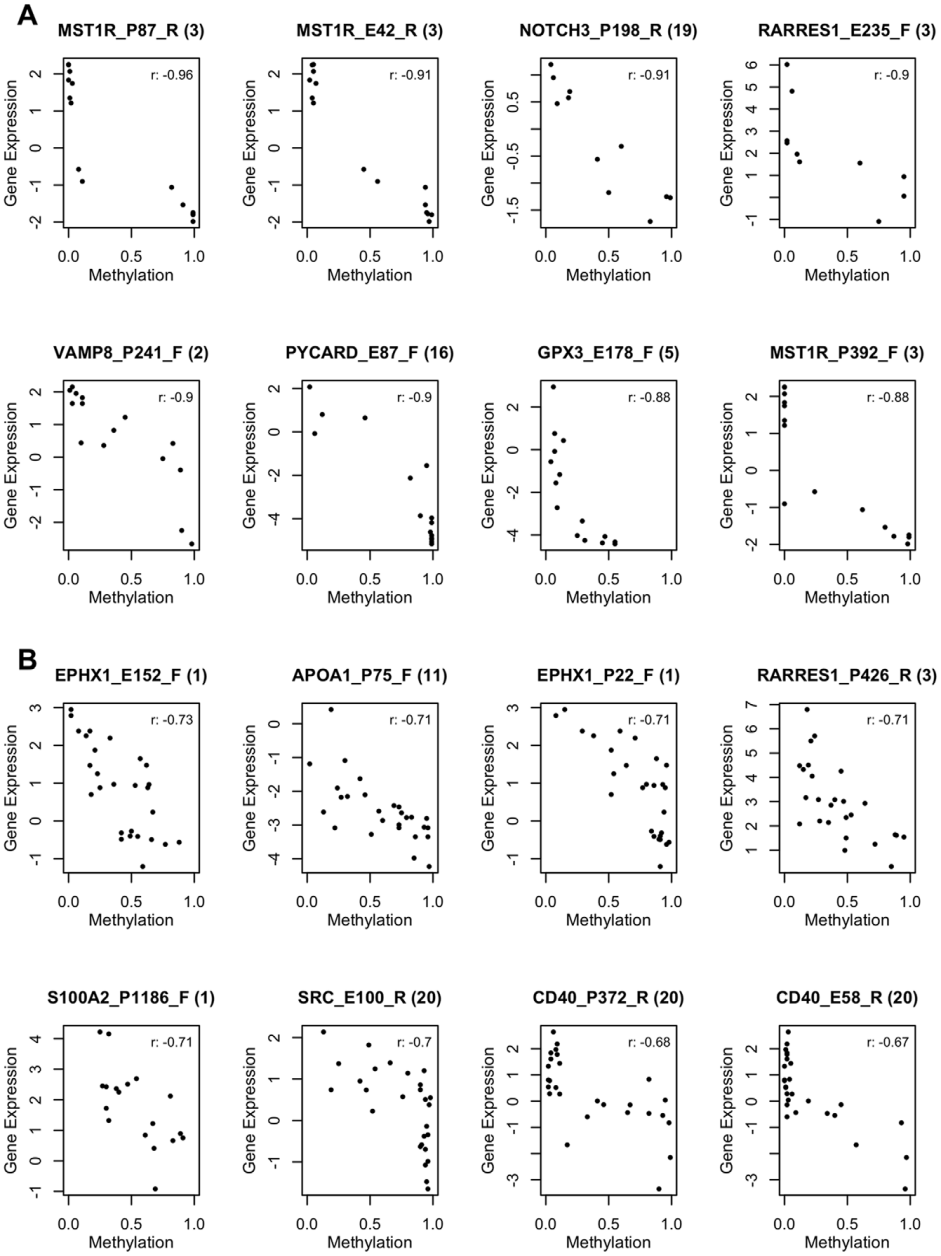
Top correlations between DNA methylation and gene expression. The 8 CpG sites with the most negative correlations between DNA methylation and gene expression levels are shown for each of the two specimen sets: A) Cell lines; B) Tumors.

To investigate the relationship between DNA methylation status and gene expression, we measured levels of transcripts from the same set of genes in the same cell lines and primary tumors, using HEEBO, a 70mer oligonucleotide DNA microarray [25] **.** We analyzed the cell line and tumor data sets separately, and in each case restricted the analysis to autosomal genes selected for variation in methylation levels across the specimen set (see Methods). Promoter DNA methylation and levels of the corresponding gene's transcript have been found to be inversely correlated in some cases, but the relationship is not necessarily linear. Therefore, we used Spearman correlation, rather than Pearson correlation to examine this relationship for each locus in each of the two datasets (Figure 4). In cell lines, we found a clear trend towards a negative correlation between DNA methylation levels and gene expression for the 828 CpG sites whose methylation levels varied among the cell lines (Figure 4A, mean ρ = −0.22). We estimated that for at least 27% of these 828 CpG sites, increases in methylation were accompanied by decreases in transcription of the associated gene (Figure 4A). For the tumor tissue samples, the correlation between DNA methylation levels and the corresponding transcript abundance, for the 758 CpG sites with significant variation in methylation across tumors, was also negative, but much weaker (Figure 4B, mean ρ = −0.08). We estimated that for at least 16% of these 758 CpG sites, increases in methylation were accompanied by decreases in transcription of the associated gene (Figure 4B). As expected, CpG sites at which methylation was positively correlated with expression of the corresponding transcript were rare. Indeed, the proportion of CpG sites exhibiting positive correlation (ρ>0.5) between methylation values and expression values was significantly less than that observed in randomly paired CpG sites (in the data from ovarian cancer cell lines, approximately 42% fewer than would be expected if there were no interaction). Scatterplots of DNA methylation levels versus gene expression levels for the 8 CpG sites with the strongest negative correlations between these values are shown in Figure 5A and 5B, for cell lines and tumors, respectively. Notably, the association between methylation of a given CpG and transcription of the corresponding gene across the cell lines was only weakly correlated to the same association across the tumors, (Pearson r = 0.19) (Supplemental Figure S4).

Hypermethylation of CpG dinucleotides located in promoter 5′CpG islands has been shown to be associated with gene silencing [26]–[29]. We therefore examined the relationship between promoter DNA hypermethylation and gene expression in the context of CpG islands (as defined according to either the Takai and Jones criteria [30] or the Gardiner-Garden criteria [31]). In the cell lines, we found that the correlation between DNA methylation and gene expression levels tended to be more strongly negative among CpGs contained within CpG islands (mean β-values: −0.25 versus −0.12 for loci that met one or more CpG island criteria (n = 585) versus loci that met zero criteria respectively (n = 243), KS test p-value <0.001). We estimated that for at least 29% of the 585 loci meeting the CpG island criteria, increases in methylation were accompanied by decreases in transcription of the associated gene (Figure 4C). The difference in correlations in CpG island loci versus non-CpG island loci was not borne out in the tumor dataset (mean β-values: −0.08 versus −0.10 for loci that met one or more CpG islands criteria versus those that met zero, KS test p-value = 0.052). In tumors, we estimated that for at least 10% of the 492 loci meeting the CpG island criteria, increases in methylation were accompanied by decreases in transcription of the associated gene (Figure 4D). We found no consistent relationship between the distance of a methylation locus from the transcription start site and the strength of the correlation between its methylation levels and expression levels of the associated gene (not shown).

## Discussion

We quantitatively assessed the DNA methylation status of 1,505 CpG sites, (associated with 808 genes), in 15 commonly used ovarian cancer cell lines and 27 primary ovarian tumors, 15 serous (55.5%), 9 endometrioid (33.3%) and 3 clear cell (11.1%). These proportions roughly reflect the relative frequencies of the different histologies of ovarian carcinoma diagnosed in the US (serous tumors 50%, endometrioid 25–30%, mucinous 10–15%, clear cell 5%, and others <5%) [32].

We found that the DNA methylation profiles of the ovarian cancer derived cell lines were distinctly different from those of the primary ovarian tumors. These results are consistent with reports describing vastly different gene expression patterns between cell lines and primary tumors in ovarian and other cancers [33]–[35]. While cell lines can be useful for molecular mechanistic studies, this study reinforces the need for skepticism in considering them as models for the cancers from which they originated, both in general and in the specific case of epigenetic profiling of ovarian cancer. One notable general difference between cell lines and tumors was the tendency for DNA methylation measurements to be higher in the cell lines than in the tumors. These findings are consistent with reports of an enhanced hypermethylation phenotype in cancer cell lines, compared to primary tumors for other types of cancer [36]. One prior study of ovarian cancer found fewer hypermethylated CpG islands in cell lines versus primary tumors [37]. Our results do not support this finding. Because the methylation sites represented in the arrays used for this study were biased towards sites known to be methylated in some cancers, this finding may be consequence of the fact that cell lines represent a ‘pure’ culture of cancerous cells whereas the primary tumors inevitably contained a mixture of tumor cells and other cells. This ‘normal contamination’ effect is supported by our finding that nearly all of the CpG sites that showed higher methylation in tumors than in cell lines also had elevated levels of DNA methylation in leukocytes from healthy women. Accumulation of epigenetic changes during prolonged cell culture may also contribute to the higher frequency of hypermethylation in the cell lines. This was observed for cancer cell lines [36], as well as hES cell lines and their derivatives [38], [39]. Furthermore, DNA methylation profiles of the hESC-derived neural precursor cells [38], [39] and hESC-derived liver cells [40] were shown to be different from their in vivo derived counterparts.

Although the numbers of some subtypes, such as clear cell tumors, were small, unsupervised and supervised analyses of the DNA methylation profiles of the primary tumors indicate clear differences between the histological types. We identified 90 CpG sites (associated with 68 genes) with significantly subtype-specific methylation patterns, the majority of which distinguished serous from endometrioid tumors. The distinctness of the DNA methylation profiles of the histological subtypes is suggestive of different tumorigenic mechanisms and/or cells of origin and underscores the need to view the different histological types of ovarian cancer as different diseases. While this notion is common knowledge among clinicians, and is reflected in studies of tumor gene expression [41] and protein expression [42], regrettably, it is often ignored in molecular diagnostic studies, and all cases of ovarian cancer are still treated similarly in clinic despite their acknowledged differences in pathology. Successful detection and treatment strategies for ovarian cancer are likely to be histology-specific, and will require a better understanding of the biological characteristics of each subtype.

As one of the largest studies to examine correlations between gene expression and DNA methylation, our results in this regard are noteworthy. We found for some genes that increased DNA methylation was correlated with decreased levels of transcription of the associated gene, but that there were also many genes for which such a relationship was not evident. We estimated that increases in methylation levels were associated with decreases in transcription of the corresponding gene for 27% of the 828 CpG sites with variable DNA methylation across 15 ovarian cancer cell lines (Figure 4A). Among loci in CpG islands, we estimated that increases in DNA methylation were accompanied by decreases in transcription of the associated gene for at least 29% of 585 CpG sites across cell lines (Figure 4C). Across all CpGs and CpG island loci, the association between DNA methylation and gene expression was much stronger among the cell lines than among the primary tumor tissues, most likely due to the confounding effect of cellular heterogeneity in the tumor specimens. Our findings are consistent with a recent study of large B-cell lymphoma [43], in which a wide range of correlations between gene expression and methylation were reported. Interestingly, all three of the genes evaluated in this study that were highlighted in the previous study as having a strong correlation between hypermethylation and expression showed a similar negative correlation in our cell line dataset (MGMT: −0.63, RBP1: −0.78, IGSF4: −0.69). Our results support the generality of the recognized relationship between promoter CpG island hypermethylation and gene silencing. However, the fact that this inverse relationship was not observed for the majority of loci, similar to another report [43], further highlights the complexity of this relationship.

The data presented here provide a first glimpse of the extent of variation in levels of DNA methylation among ovarian cancer specimens and as such provide a useful new lens through which to examine their defining characteristics. In principal, DNA methylation profiling could also be useful for discovery of biomarkers for detection of ovarian cancer, based on assays using sensitive PCR-based approaches for detection of methylated DNA [44]–[51]. However, even if we can identify a DNA-methylation marker that distinguishes tumors from normal cells with high sensitivity and specificity, additional criteria would need to be satisfied in order for this approach to be viable: adequate quantities of tumor-derived DNA would need to reach and persist in an assayable fluid (like blood or proximal fluids) in women with occult ovarian cancers still at a curable stage, and the abundance of the specifically methylated DNA sequences in fluids from these women would need to significantly exceed background levels in cancer-free individuals. These criteria seem difficult to satisfy in a blood-based early detection test for ovarian cancer, given the small size of the tumors that we need to detect [52], [53]. To this end, and as a primary step, we compared the DNA methylation profiles of normal leukocytes and ovarian tumors. But the potential of DNA methylation markers as early-detection markers using proximal fluids or for detection of recurrent cancer in a blood-based assay remains to be evaluated. Additional profiling of primary tumors and corresponding normal tissues (fallopian tube, ovarian surface epithelium, peritoneum or endometrium) as well as measurements of methylated DNA in candidate detection media (blood or proximal fluids) in both health and disease will help define the natural history of ovarian cancers and critically assess the potential utility of DNA methylation based biomarkers in combating ovarian cancer.

## Materials and Methods

### Sample Collection and Processing

#### Ovarian tumors

Tumor tissues were obtained from patients with informed consent, which was approved by the Institutional Review Board of the Swedish Hospital in Seattle. Tissue acquisition, processing and storage were conducted by the Pacific Ovarian Cancer Research Consortium (POCRC) at the Fred Hutchinson Cancer Research Center. The quality of tumor tissues, the percentage of cancer cells and the histological analysis of the tumors were determined via a centralized pathology review at the POCRC repository. All samples were distributed in a de-identified manner and cannot be traced back to patients. Tumor and patient characteristics are summarized in Table 1.

#### Cell lines

The sources and characteristics of the 15 ovarian cancer-derived cell lines analyzed are summarized in Table 2. All cell lines were cultured according to the specifications outlined by ATCC. Cell lines were grown to 80% confluence, then serum-deprived in 0.5% FBS for 24 hours before harvesting. Cells were washed twice with Hank's Balanced Salt Solution (Invitrogen, Carlsbad, California) and drained. 1 mL of SDS extraction buffer (0.1M NaCl, 20 mM Trizma base, 25 mM EDTA, 0.5% w/v SDS) was added directly to the plate to lyse the cells. Cellular lysate was scraped off the plates, pipetted into a cryovial, and stored at −80°C until DNA extraction. Lysates were treated with 200 ug/ml ProteinaseK at 50°C overnight. DNA was precipitated with one volume of Isopropanol and dissolved in TE^−4^ buffer.

#### Buffy coat

Whole blood from two females (age>60) was purchased from HemaCare Corporation. Blood was collected in anticoagulant containing blood collection bags and kept on ice. Samples were processed immediately upon receipt.

#### DNA and RNA extraction from ovarian tumors

The tumor tissues were disrupted in a Biospec Tissue-Tearor Model 985370-395 in the presence of TRIzol (Invitrogen) with a ratio of 10 ml TRIzol per 50 mg of tissue. Following an extraction step of 0.2 ml of Chloroform per 1 ml of TRIzol, the upper aqueous phase was removed for RNA isolation. The remaining inter-phase and the phenol phase was briefly stored in 4°C for DNA isolation. The upper aqueous phase was processed for total RNA according to Invitrogen protocol and further purified using RNeasy Mini kit (Qiagen). Genomic DNA was isolated by back-extraction from the TRIzol inter-phase and the phenol phase as described on Ambion website (page 4) [54]. Detailed amplification and labeling protocols are available at the Brown lab website [55].

#### DNA extraction from buffy coat

Blood samples were centrifuged for 10 min at 300 *g* in 50 ml conical tubes, followed by another 10 min centrifugation at 1,600 *g.* Buffy coat was isolated and DNA was extracted using Qiagen QIAamp DNA Blood Kit and stored in −30 C until sodium bisulfite conversion.

#### RNA extraction from ovarian cell lines

Cell lines were lysed in Phenol followed by a Phenol/Chloroform extraction. The aqueous layer was added to 70% ethanol and further purified by loading the RNA/Ethanol mix into an RNeasy Mini kit (Qiagen) column. Purification was continued and finalized as described in the RNeasy kit (Qiagen) Manual. Detailed amplification and labeling protocols are available at the Brown lab website [55].

### DNA Methylation Analysis

#### DNA methylation assay platform

DNA methylation analysis was performed using Illumina's Golden Gate Cancer Panel 1 bead array [16]. These arrays contain DNA probes representing 1505 CpG islands near 808 genes. Probes were selected for cancer-related genes based on data from Illumina's scientific collaborators, literature, and public databases.

#### Experimental methods

Briefly, genomic DNA from 27 primary ovarian tumors and 15 ovarian cell lines was subject to sodium bisulfite conversion, labeled with fluorescent dyes, and hybridized to Illumina Golden Gate Cancer Panel 1 bead arrays at the University of Southern California Epigenome Center. We utilized a number of DNA methylation control reactions to assess the extent of bisulfile conversion completion as explained in detail elsewhere [56] **.** Methods and reagents for sodium bisulfite conversion and array hybridizations were as previously described [8], [16], [57].

#### Data processing

DNA methylation values from the bead array hybridizations were scored as β-values [16]. β-values for each probe in each experiment were calculated as the ratio of the methylated signal over the total fluorescent signal on a scale between 0 and 1 [16]. β-values thus indicate a locus' observed methylation relative to the maximum potential methylation of that site. For quality control, we obtained non-background corrected signal intensities of the methylated (M) and unmethylated (U) probes and the mean negative control cy5 (red) and cy3 (green) signal intensities. We also obtained the Detection p-values, which provide an indication of DNA methylation measurement quality for each locus and are calculated based on the difference in signal intensity of each probe compared to the set of negative control probes. Measurements for which the detection p-value is less than 0.05 are considered to have a signal intensity significantly above background. Data points with a detection p-value>0.05 are masked as “NA”, and represent beta values with non-significant detection of DNA methylation compared to background.

### Gene Expression Analysis

#### Assay platform

We measured transcript levels in the 27 primary ovarian tumors and 15 cell lines using the HEEBO [16] oligonucleotide microarrays, containing 44,544 70-mer probes and printed at the Stanford Functional Genomics Facility. Detailed information on the HEEBO arrays is available at the Stanford Functional Genomics Facility website [25].

#### Experimental methods

Microarray experiments were performed as described at the Brown lab website [58]. Briefly, 500 µg of total RNA from each of the ovarian tumors were amplified using Amino Allyl Message Amp™ II aRNA Kit (Ambion, Austin, TX, USA). The aRNAs were labeled with Cy5 and co-hybridized with Cy3 labeled Stratagene reference aRNA. For some samples, the mRNA was amplified and hybridized in duplicate or triplicate. The samples were then hybridized to HEEBO microarrays. The arrays were scanned in a low-ozone environment using a GenePix 4000A microarray scanner and images were analyzed with Genepix 5.0 (Axon instruments, Union City, CA). The raw data were deposited into Stanford Microarray Database [59] and can be viewed at the SMD website [60].

#### Data processing

**1) By Spot:** A spot quality filter was applied to every spot on each array. Only spots with a Cy5/Cy3 ratio of intensity/background >1.5 in either channel were included. Spots that were flagged manually for poor quality by visual inspection of spot uniformity were excluded. The log (base2) of Cy5/Cy3 normalized ratio (mean) was calculated for each spot. Ratios were normalized for each array such that the distribution of log ratios for the array had a mean of 0.0. Empty and control spots were dropped after normalization. Spots with the same Entrez Gene ID were averaged. **2) By Array:** Replicate hybridizations of the same amplified mRNA were averaged by Entrez Gene ID. Hybridizations of independent amplifications of the same sample mRNA were averaged by Entrez Gene ID.

### Statistical Methods

#### DNA methylation clustering and visualization

To visualize the DNA methylation patterns across all ovarian cancer cell lines and tumors, we first filtered for genes with some variation across the sample set. Specifically, we restricted our analysis to CpG sites with minimum β-value greater than 0.2, ratio of maximum β-value to minimum β-value greater than or equal to 2 and 1 or fewer missing β-values. We then mean-centered the selected 1184 β-values by gene and applied two-way average-linkage unsupervised hierarchical clustering on the resulting data. Note that while this data are filtered, no comparisons were made and data were clustered for both samples and CpG sites. We visualized the clusters in Java Treeview [61], [62].

#### Identification of loci with biopecimen-specific DNA methylation levels (cell line versus tumor)

To identify loci that showed significantly different levels of DNA methylation in cell lines versus tumors, we first restricted our analysis to the 1,110 autosomal CpG sites whose β-values were variable across the set of 15 cell lines and 27 tumors. We defined variable CpG sites as those CpG sites with minimum β-value greater than 0.2, ratio of maximum β-value to minimum β-value greater than or equal to 2 and 1 or fewer missing β-values. We then performed a two-sample t-test comparing the 15 cell lines to the 27 tumors. P-values from the t-test were adjusted for multiple comparisons by controlling the false discovery rate [23]. All statistical analyses were performed using the R software package [63].

#### Identification of loci with histological subtype-specific DNA methylation levels

To identify ovarian cancer subtype-specific loci, we first restricted our analysis to the 818 autosomal CpG sites whose β-values were variable among the 27 primary tumors. We defined variable CpG sites as those CpG sites with minimum β-value greater than 0.2, ratio of maximum β-value to minimum β-value greater than or equal to 2 and 1 or fewer missing β-values. We performed pair-wise two-sample t-tests comparing each subtype independently to the other subtypes (serous vs. endometrioid, serous vs. clear cell, endometrioid vs. clear cell). P-values from each pair-wise analysis were adjusted for multiple comparisons by controlling the false discovery rate [23]. All statistical analyses were performed using the R software package [63].

#### Association of DNA methylation and gene expression

We paired loci used in methylation analysis with gene expression data from the appropriate loci according to Entrez Gene ID. For gene expression data, we used the normalized log ratios of Cy5/Cy3 (see Microarray Methods). For DNA methylation, normalized β-values were used. We excluded X-linked loci from this analysis because of the X-chromosome associated dosage compensation mechanisms could cause a decoupling of methylation levels and gene expression levels. We further restricted our analysis to loci with at least 5 (of 15) non-missing gene expression measurements in the cell line analysis and 9 (of 27) non-missing gene expression measurements in the tumor analysis. For the 758 CpG sites that were sufficiently variable across all tumor specimens (see filtering above), we calculated the Spearman correlation between DNA methylation levels and gene expression levels. Similarly, we calculated Spearman correlations for the 828 CpG sites that were sufficiently variable across all cell line samples (see filtering above). For each dataset, the false discovery rate was estimated by calculating the median number of loci with correlations below a given threshold over 500 permutations of the gene labels. All statistical analyses were performed using the R software package [63].

## Supporting Information

File S1 Methylation array probe annotations and associated methylation and gene expression data. List of probes and associated annotations (locus, gene, distance from transcription start, etc) for the Illumina bead array used to profile specimens. Observed methylation values (β-values) for each probe and specimen are recorded as are the gene expression levels (log base2 ratio) of the associated transcripts.
(2.20 MB XLS)

File S2 Loci with differential DNA methylation. List of CpG sites with significant differences in DNA methylation between cell lines and tumors or between tumor histologies. Associated probe data are also included.
(0.40 MB XLS)

File S3 CpG island information and distance from the transcription start site of probes with differential methylation between tumor histologies.
(0.06 MB XLS)

Figure S1 Correlation in DNA methylation values between pairs of probes. The distribution of Pearson correlations is shown for i) pairs of related probes (2 CpG sites, 1 gene) ii) pairs of unrelated probes (2 CpG sites, 2 genes). For related probes, only the 1184 probes (686 genes) which exhibited sufficient variation across the 42 specimens (see Methods) were included.
(0.36 MB TIF)

Figure S2 Correlation matrix for DNA methylation values across loci. Pearson correlations in methylation values for pairs of CpG sites are shown colorimetrically. CpG sites are sorted by their location in the genome. The 1,184 CpG sites (686 genes) selected for variation in methylation across all specimens were used (Figure 1). Correlation calculations were based on 42 specimens. Red indicates positive correlation, green indicates negative correlation.
(5.37 MB PDF)

Figure S3 Overlap between tumor histology lists. A) Venn diagram showing number of CpG sites in each histology-specific list and overlap between lists; B) Venn diagram showing number of genes in each histology-specific list and overlap between lists. S = Serous tumor, E = Endometrioid tumor, CC = Clear cell tumor.
(0.13 MB TIF)

Figure S4 Methylation-gene expression correlation across cell lines versus methylation-gene expression correlation across tumors.
(0.17 MB TIF)

Appendix S1 Pacific Ovarian Cancer Research Consortium Menopausal Determination
(0.02 MB DOC)
