# Supplementary figures and images for: DNA Methylation Profiles of Ovarian Epithelial Carcinoma Tumors and Cell Lines
Source: PLoS One. 2010 Feb 22;5(2):e9359. doi: 10.1371/journal.pone.0009359 (PMC2825254; doi:10.1371/journal.pone.0009359)

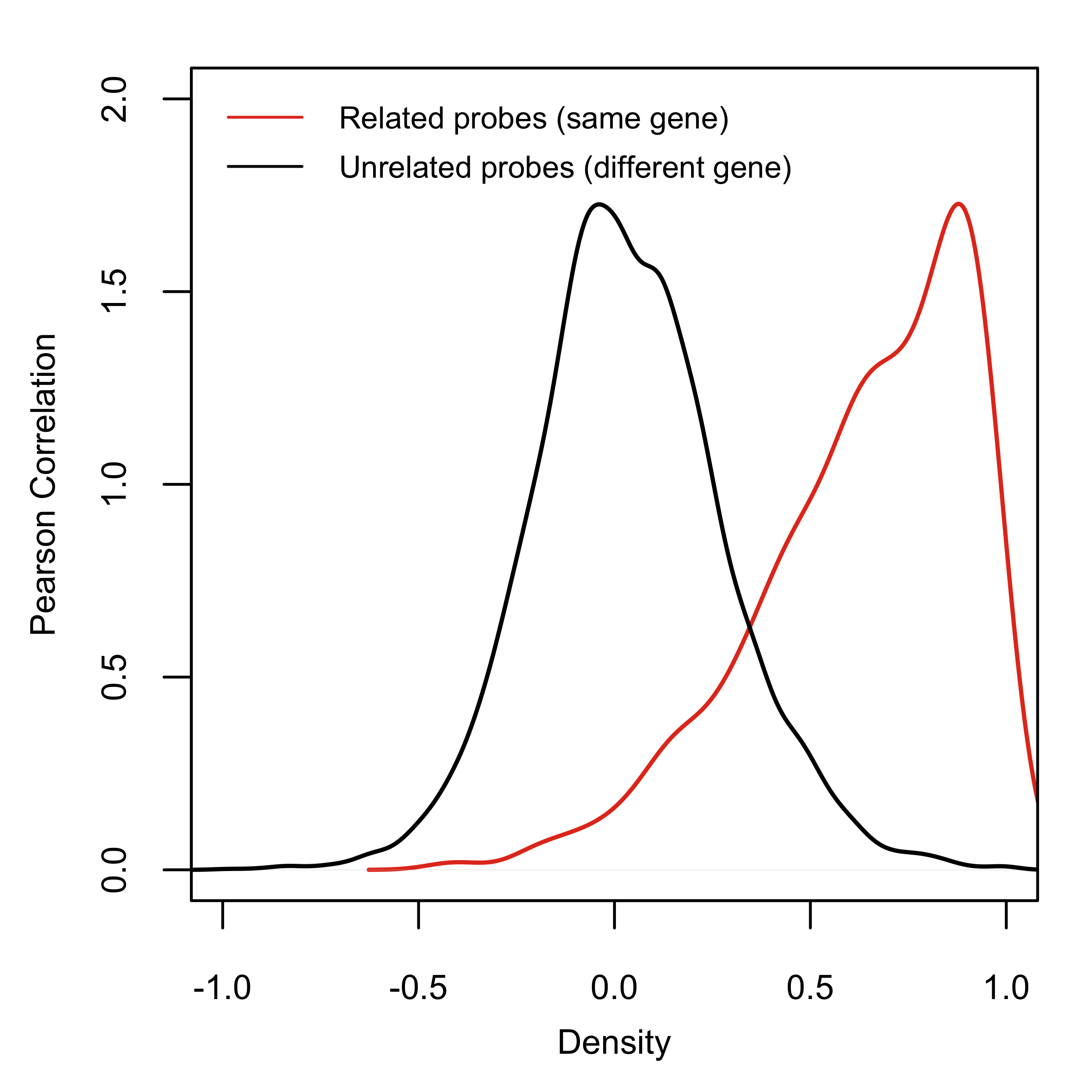

Supplement: Figure S1 — Correlation in DNA methylation values between pairs of probes. The distribution of Pearson correlations is shown for i) pairs of related probes (2 CpG sites, 1 gene) ii) pairs of unrelated probes (2 CpG sites, 2 genes). For related probes, only the 1184 probes (686 genes) which exhibited sufficient variation across the 42 specimens (see Methods) were included. (0.36 MB TIF) [file pone.0009359.s004.tif]

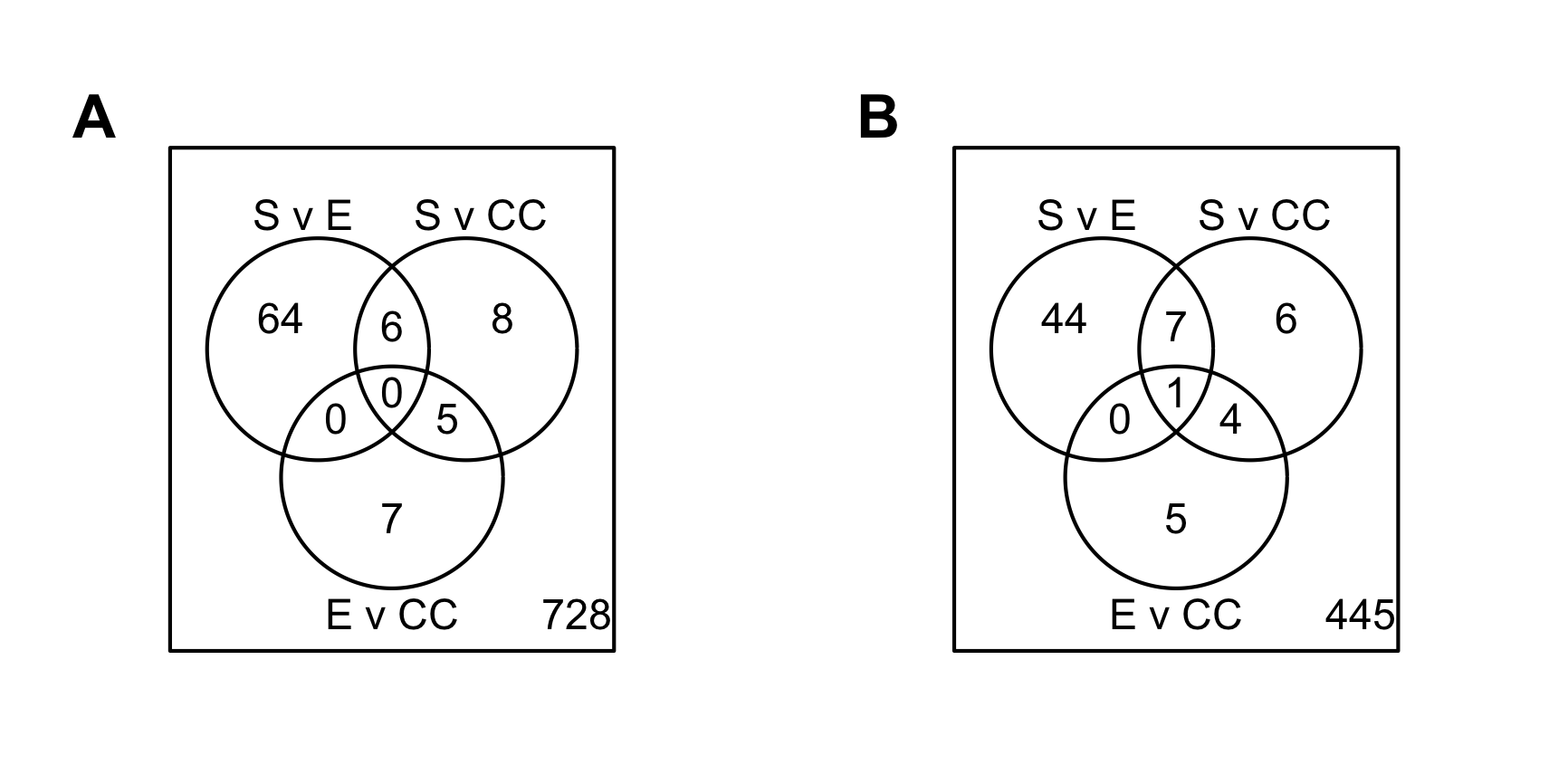

Supplement: Figure S3 — Overlap between tumor histology lists. A) Venn diagram showing number of CpG sites in each histology-specific list and overlap between lists; B) Venn diagram showing number of genes in each histology-specific list and overlap between lists. S = Serous tumor, E = Endometrioid tumor, CC = Clear cell tumor. (0.13 MB TIF) [file pone.0009359.s006.tif]

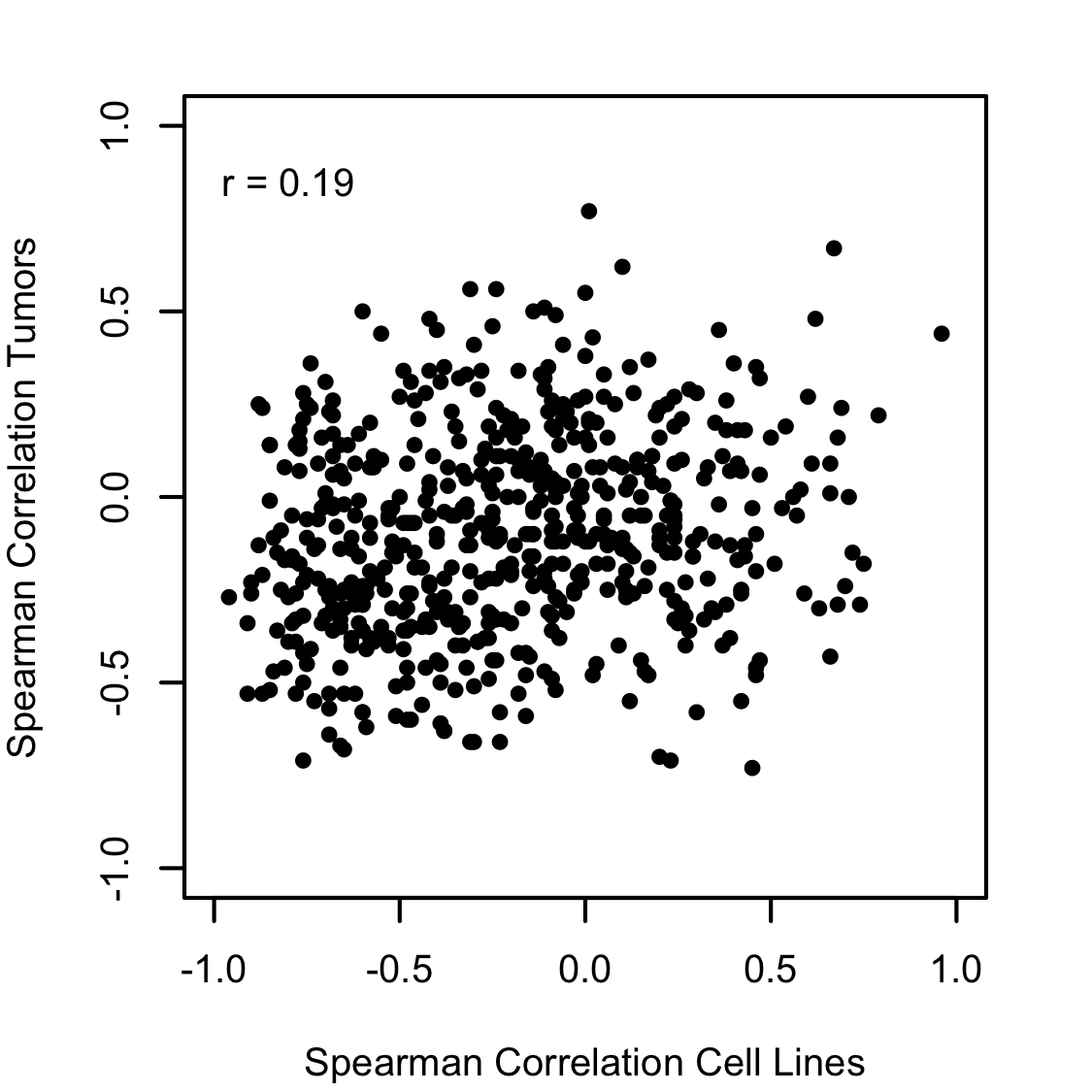

Supplement: Figure S4 — Methylation-gene expression correlation across cell lines versus methylation-gene expression correlation across tumors. (0.17 MB TIF) [file pone.0009359.s007.tif]
