# Supplementary material for: DNA Methylation Profiles of Ovarian Epithelial Carcinoma Tumors and Cell Lines
Source: PLoS One. 2010 Feb 22;5(2):e9359. doi: 10.1371/journal.pone.0009359 (PMC2825254; doi:10.1371/journal.pone.0009359)
Supplement: Appendix S1 — Pacific Ovarian Cancer Research Consortium Menopausal Determination (0.02 MB DOC) [file pone.0009359.s008.doc]

Age 55+ = menopausal

Age 50+ = menopausal if menstruating and on HRT

Age <55 = pre-menopausal if menstruating

Age <55 = peri-menopausal if menstruation in the last year, but not in the last 3 months

Age <50 = pre-menopausal if hysterectomy and at least one ovary

Age 50+ = menopausal if ovaries, but no uterus

Menopausal if ovaries and uterus, but no menstruation in the last year
